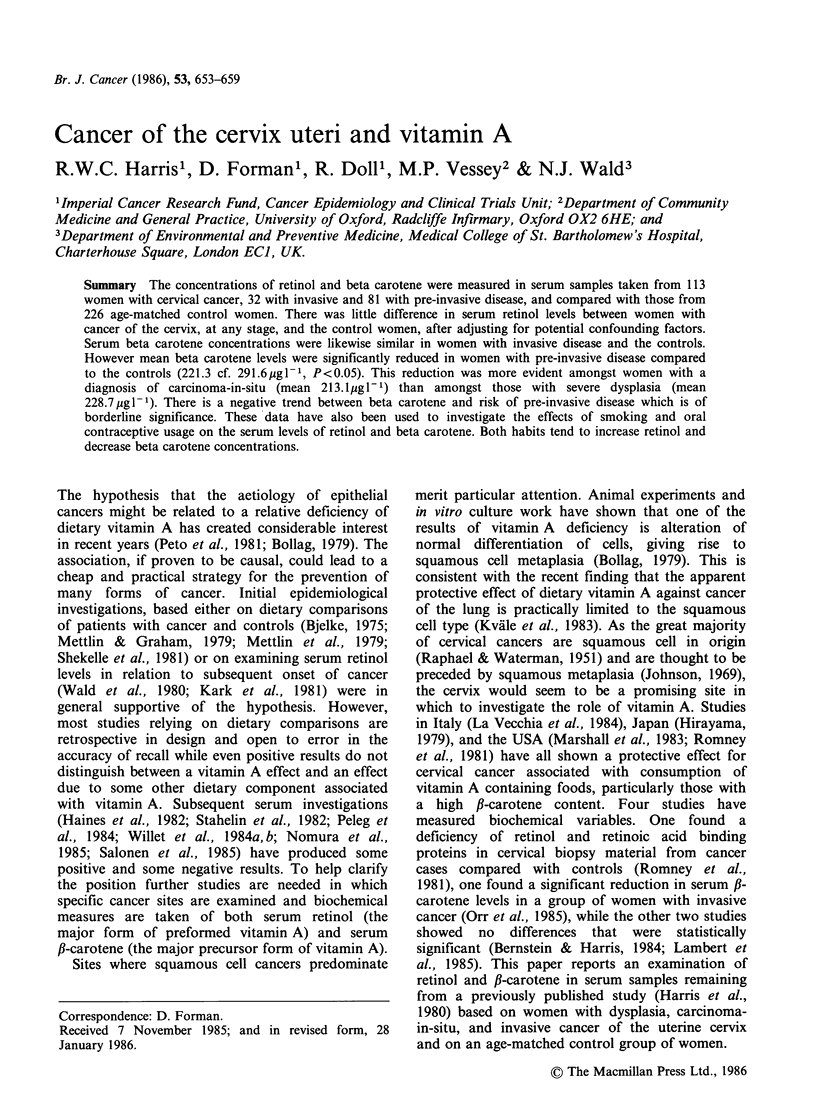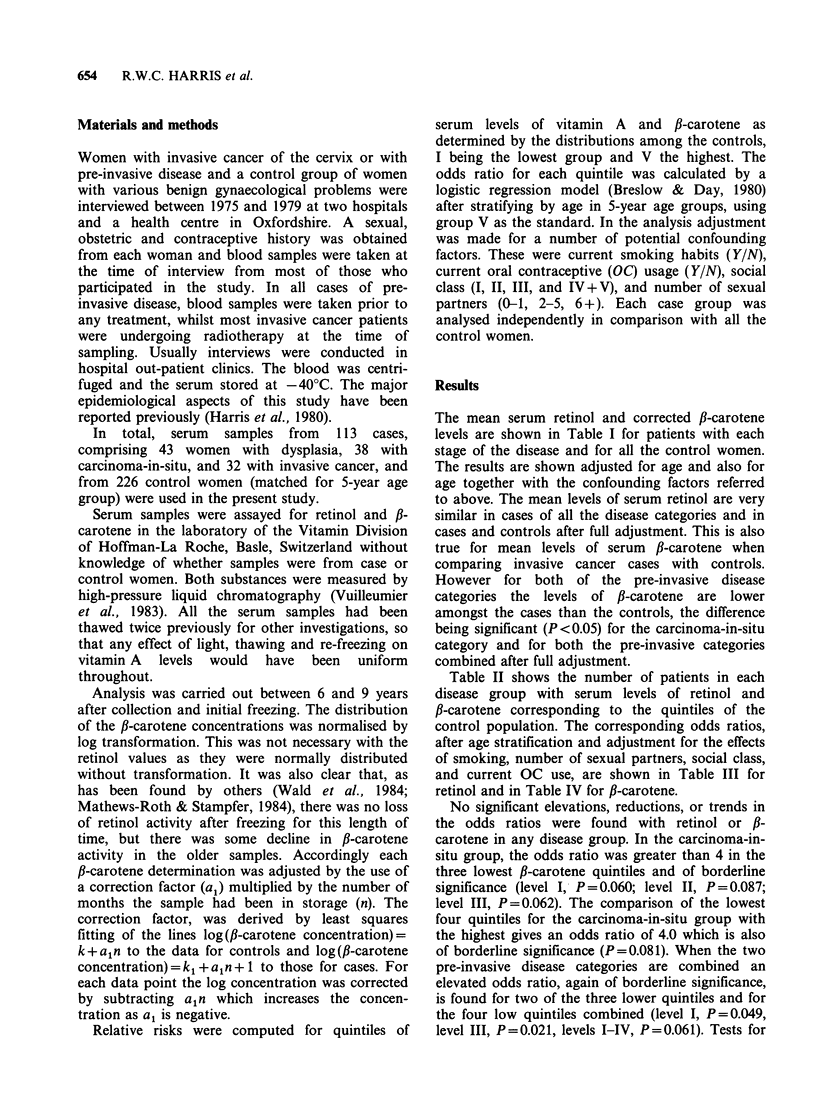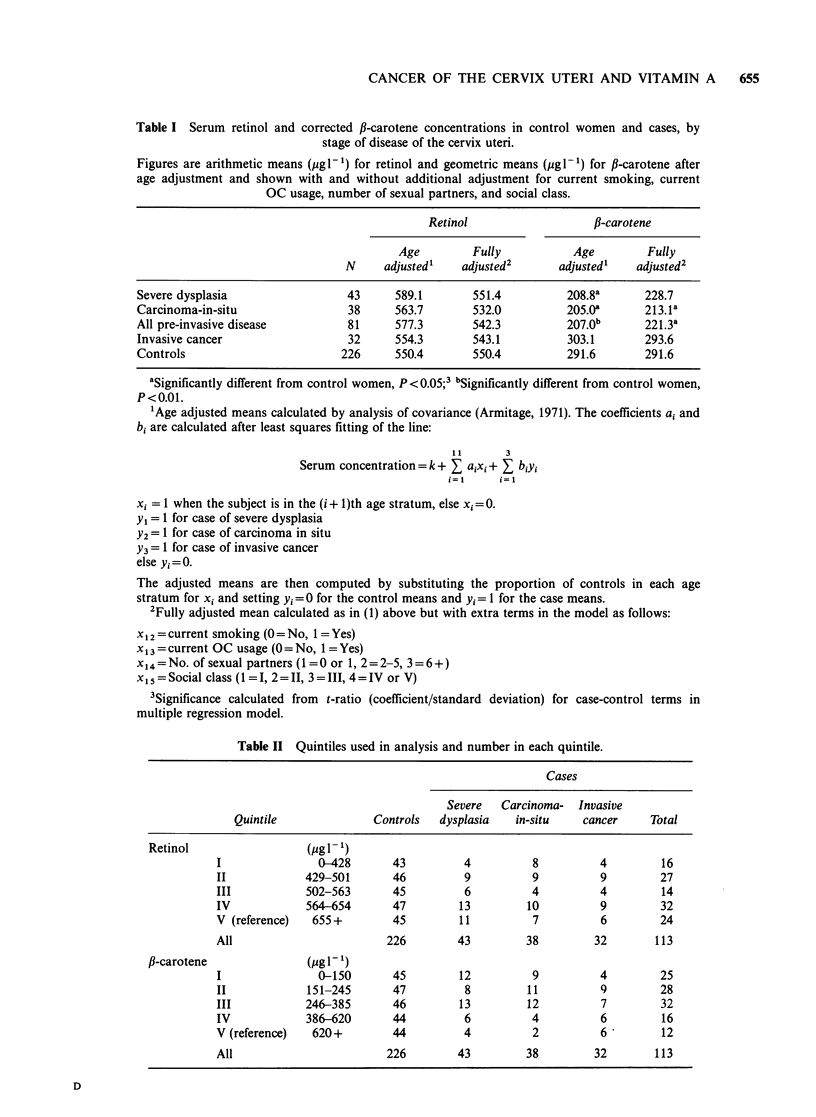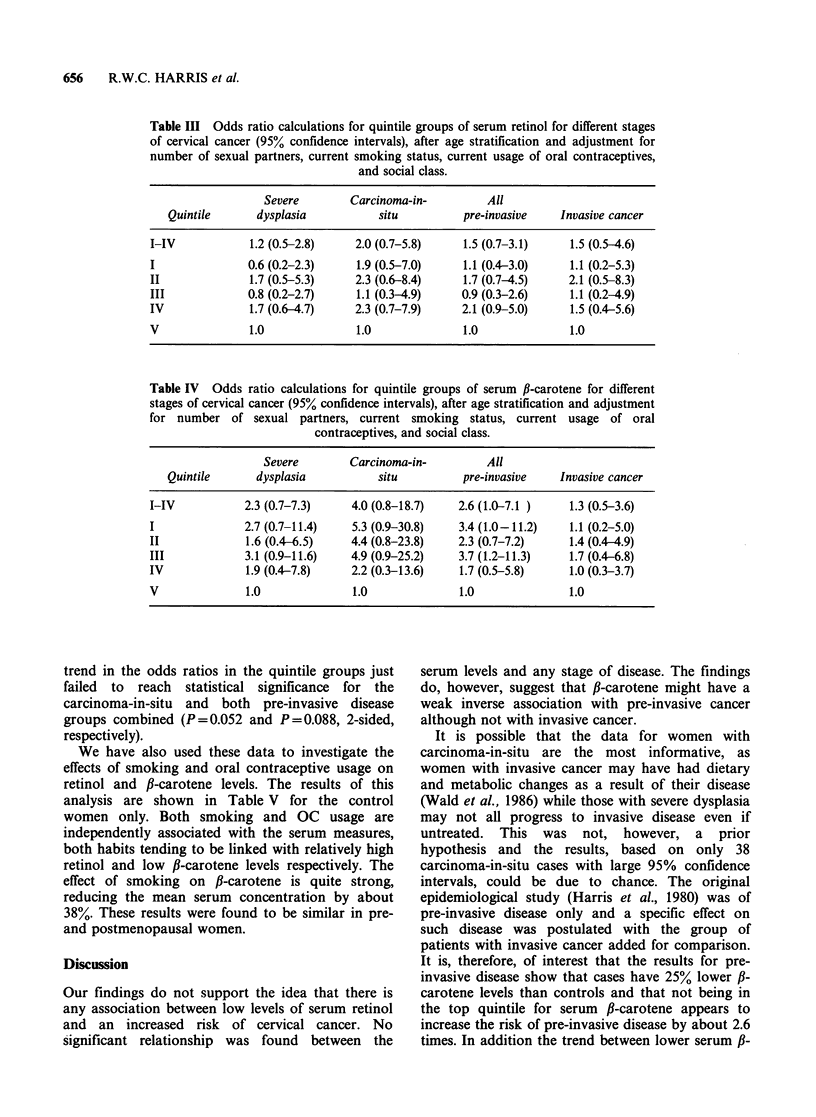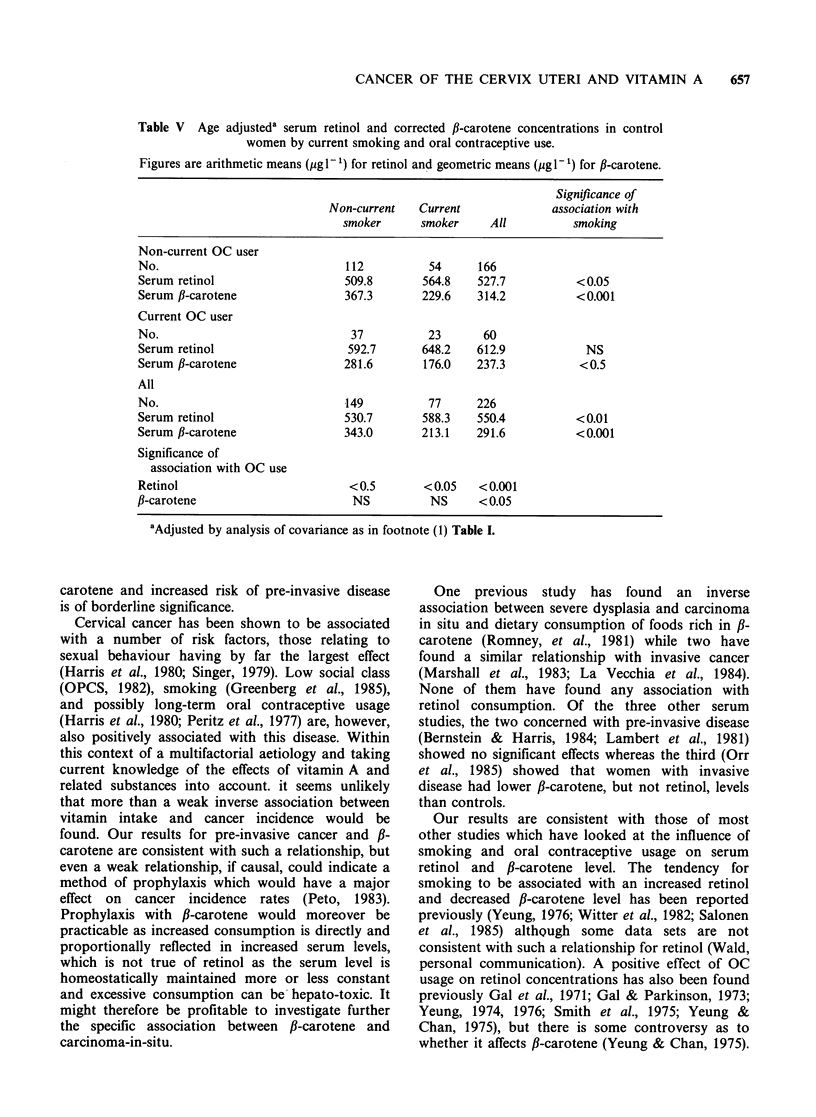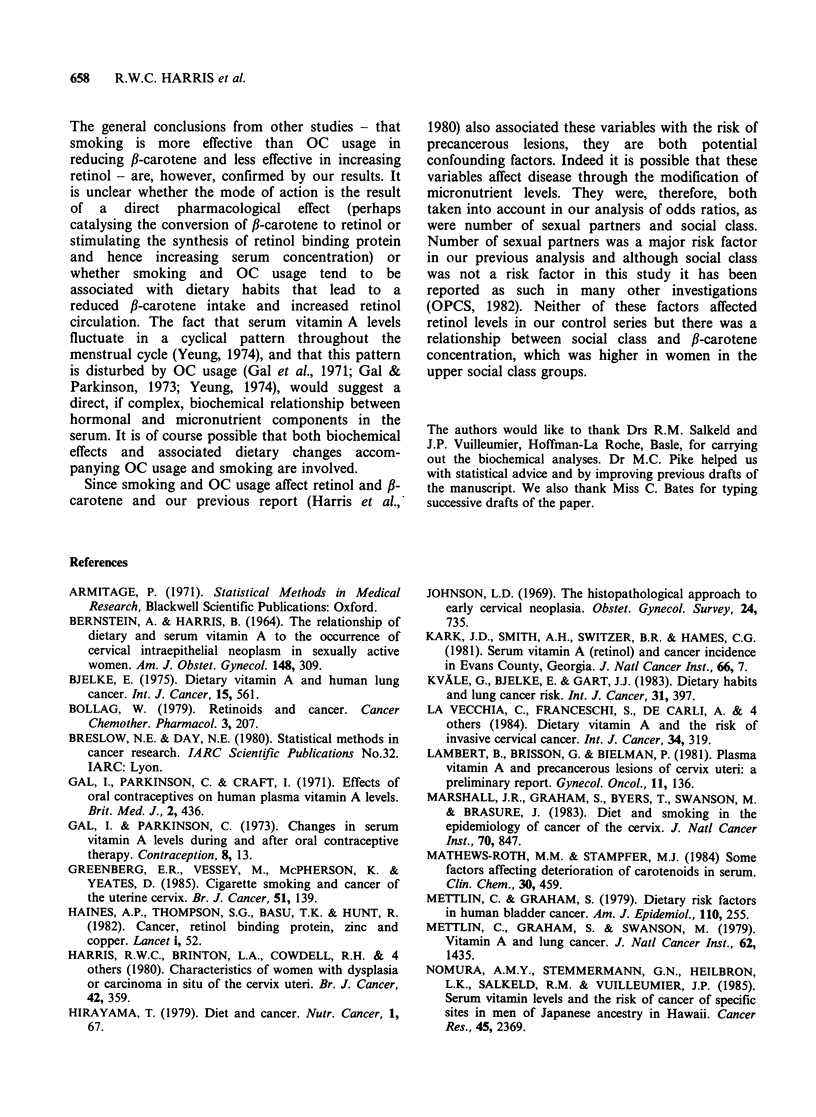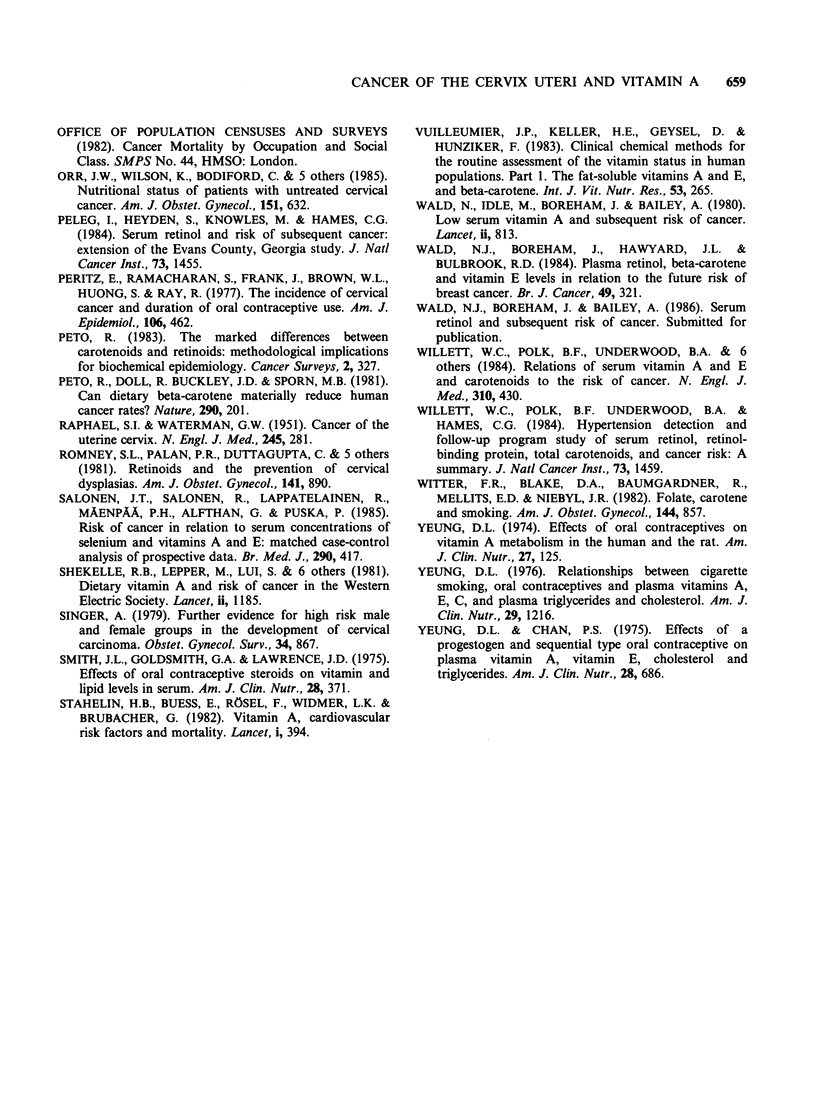# Cancer of the cervix uteri and vitamin A.

**DOI:** 10.1038/bjc.1986.109

**Published:** 1986-05

**Authors:** R. W. Harris, D. Forman, R. Doll, M. P. Vessey, N. J. Wald

## Abstract

The concentrations of retinol and beta carotene were measured in serum samples taken from 113 women with cervical cancer, 32 with invasive and 81 with pre-invasive disease, and compared with those from 226 age-matched control women. There was little difference in serum retinol levels between women with cancer of the cervix, at any stage, and the control women, after adjusting for potential confounding factors. Serum beta carotene concentrations were likewise similar in women with invasive disease and the controls. However mean beta carotene levels were significantly reduced in women with pre-invasive disease compared to the controls (221.3 cf. 291.6 micrograms l-1, P less than 0.05). This reduction was more evident amongst women with a diagnosis of carcinoma-in-situ (mean 213.1 micrograms l-1 than amongst those with severe dysplasia (mean 228.7 micrograms l-1. There is a negative trend between beta carotene and risk of pre-invasive disease which is of borderline significance. These data have also been used to investigate the effects of smoking and oral contraceptive usage on the serum levels of retinol and beta carotene. Both habits tend to increase retinol and decrease beta carotene concentrations.


*Br. J. Cancer* (1986), **53**, 653–659

### Cancer of the cervix uteri and vitamin A

R.W.C. Harris[1], D. Forman[1], R. Doll[1], M.P. Vessey[2] & N.J. Wald[3]

[1]*Imperial Cancer Research Fund, Cancer Epidemiology and Clinical Trials Unit;* [2]*Department of Community Medicine and General Practice, University of Oxford, Radcliffe Infirmary, Oxford OX2 6HE; and* [3]*Department of Environmental and Preventive Medicine, Medical College of St. Bartholomew's Hospital, Charterhouse Square, London EC1, UK.*

**Summary** The concentrations of retinol and beta carotene were measured in serum samples taken from 113 women with cervical cancer, 32 with invasive and 81 with pre-invasive disease, and compared with those from 226 age-matched control women. There was little difference in serum retinol levels between women with cancer of the cervix, at any stage, and the control women, after adjusting for potential confounding factors. Serum beta carotene concentrations were likewise similar in women with invasive disease and the controls. However mean beta carotene levels were significantly reduced in women with pre-invasive disease compared to the controls (221.3 cf. 291.6 $\mu g l^{-1}$, $P<0.05$). This reduction was more evident amongst women with a diagnosis of carcinoma-in-situ (mean 213.1 $\mu g l^{-1}$) than amongst those with severe dysplasia (mean 228.7 $\mu g l^{-1}$). There is a negative trend between beta carotene and risk of pre-invasive disease which is of borderline significance. These data have also been used to investigate the effects of smoking and oral contraceptive usage on the serum levels of retinol and beta carotene. Both habits tend to increase retinol and decrease beta carotene concentrations.

The hypothesis that the aetiology of epithelial cancers might be related to a relative deficiency of dietary vitamin A has created considerable interest in recent years (Peto *et al.*, 1981; Bollag, 1979). The association, if proven to be causal, could lead to a cheap and practical strategy for the prevention of many forms of cancer. Initial epidemiological investigations, based either on dietary comparisons of patients with cancer and controls (Bjelke, 1975; Mettlin & Graham, 1979; Mettlin *et al.*, 1979; Shekelle *et al.*, 1981) or on examining serum retinol levels in relation to subsequent onset of cancer (Wald *et al.*, 1980; Kark *et al.*, 1981) were in general supportive of the hypothesis. However, most studies relying on dietary comparisons are retrospective in design and open to error in the accuracy of recall while even positive results do not distinguish between a vitamin A effect and an effect due to some other dietary component associated with vitamin A. Subsequent serum investigations (Haines *et al.*, 1982; Stahelin *et al.*, 1982; Peleg *et al.*, 1984; Willet *et al.*, 1984*a,b*; Nomura *et al.*, 1985; Salonen *et al.*, 1985) have produced some positive and some negative results. To help clarify the position further studies are needed in which specific cancer sites are examined and biochemical measures are taken of both serum retinol (the major form of preformed vitamin A) and serum $\beta$-carotene (the major precursor form of vitamin A).

Sites where squamous cell cancers predominate merit particular attention. Animal experiments and *in vitro* culture work have shown that one of the results of vitamin A deficiency is alteration of normal differentiation of cells, giving rise to squamous cell metaplasia (Bollag, 1979). This is consistent with the recent finding that the apparent protective effect of dietary vitamin A against cancer of the lung is practically limited to the squamous cell type (Kväle *et al.*, 1983). As the great majority of cervical cancers are squamous cell in origin (Raphael & Waterman, 1951) and are thought to be preceded by squamous metaplasia (Johnson, 1969), the cervix would seem to be a promising site in which to investigate the role of vitamin A. Studies in Italy (La Vecchia *et al.*, 1984), Japan (Hirayama, 1979), and the USA (Marshall *et al.*, 1983; Romney *et al.*, 1981) have all shown a protective effect for cervical cancer associated with consumption of vitamin A containing foods, particularly those with a high $\beta$-carotene content. Four studies have measured biochemical variables. One found a deficiency of retinol and retinoic acid binding proteins in cervical biopsy material from cancer cases compared with controls (Romney *et al.*, 1981), one found a significant reduction in serum $\beta$-carotene levels in a group of women with invasive cancer (Orr *et al.*, 1985), while the other two studies showed no differences that were statistically significant (Bernstein & Harris, 1984; Lambert *et al.*, 1985). This paper reports an examination of retinol and $\beta$-carotene in serum samples remaining from a previously published study (Harris *et al.*, 1980) based on women with dysplasia, carcinoma-in-situ, and invasive cancer of the uterine cervix and on an age-matched control group of women.

Correspondence: D. Forman.
Received 7 November 1985; and in revised form, 28 January 1986.

© The Macmillan Press Ltd., 1986

### Materials and methods

Women with invasive cancer of the cervix or with pre-invasive disease and a control group of women with various benign gynaecological problems were interviewed between 1975 and 1979 at two hospitals and a health centre in Oxfordshire. A sexual, obstetric and contraceptive history was obtained from each woman and blood samples were taken at the time of interview from most of those who participated in the study. In all cases of pre-invasive disease, blood samples were taken prior to any treatment, whilst most invasive cancer patients were undergoing radiotherapy at the time of sampling. Usually interviews were conducted in hospital out-patient clinics. The blood was centrifuged and the serum stored at −40°C. The major epidemiological aspects of this study have been reported previously (Harris *et al.*, 1980).

In total, serum samples from 113 cases, comprising 43 women with dysplasia, 38 with carcinoma-in-situ, and 32 with invasive cancer, and from 226 control women (matched for 5-year age group) were used in the present study.

Serum samples were assayed for retinol and β-carotene in the laboratory of the Vitamin Division of Hoffman-La Roche, Basle, Switzerland without knowledge of whether samples were from case or control women. Both substances were measured by high-pressure liquid chromatography (Vuilleumier *et al.*, 1983). All the serum samples had been thawed twice previously for other investigations, so that any effect of light, thawing and re-freezing on vitamin A levels would have been uniform throughout.

Analysis was carried out between 6 and 9 years after collection and initial freezing. The distribution of the β-carotene concentrations was normalised by log transformation. This was not necessary with the retinol values as they were normally distributed without transformation. It was also clear that, as has been found by others (Wald *et al.*, 1984; Mathews-Roth & Stampfer, 1984), there was no loss of retinol activity after freezing for this length of time, but there was some decline in β-carotene activity in the older samples. Accordingly each β-carotene determination was adjusted by the use of a correction factor ($a_1$) multiplied by the number of months the sample had been in storage ($n$). The correction factor, was derived by least squares fitting of the lines log(β-carotene concentration) = $k+a_1n$ to the data for controls and log(β-carotene concentration) = $k_1+a_1n+1$ to those for cases. For each data point the log concentration was corrected by subtracting $a_1n$ which increases the concentration as $a_1$ is negative.

Relative risks were computed for quintiles of serum levels of vitamin A and β-carotene as determined by the distributions among the controls, I being the lowest group and V the highest. The odds ratio for each quintile was calculated by a logistic regression model (Breslow & Day, 1980) after stratifying by age in 5-year age groups, using group V as the standard. In the analysis adjustment was made for a number of potential confounding factors. These were current smoking habits ($Y/N$), current oral contraceptive ($OC$) usage ($Y/N$), social class (I, II, III, and IV + V), and number of sexual partners (0–1, 2–5, 6+). Each case group was analysed independently in comparison with all the control women.

### Results

The mean serum retinol and corrected β-carotene levels are shown in Table I for patients with each stage of the disease and for all the control women. The results are shown adjusted for age and also for age together with the confounding factors referred to above. The mean levels of serum retinol are very similar in cases of all the disease categories and in cases and controls after full adjustment. This is also true for mean levels of serum β-carotene when comparing invasive cancer cases with controls. However for both of the pre-invasive disease categories the levels of β-carotene are lower amongst the cases than the controls, the difference being significant ($P<0.05$) for the carcinoma-in-situ category and for both the pre-invasive categories combined after full adjustment.

Table II shows the number of patients in each disease group with serum levels of retinol and β-carotene corresponding to the quintiles of the control population. The corresponding odds ratios, after age stratification and adjustment for the effects of smoking, number of sexual partners, social class, and current OC use, are shown in Table III for retinol and in Table IV for β-carotene.

No significant elevations, reductions, or trends in the odds ratios were found with retinol or β-carotene in any disease group. In the carcinoma-in-situ group, the odds ratio was greater than 4 in the three lowest β-carotene quintiles and of borderline significance (level I, $P=0.060$; level II, $P=0.087$; level III, $P=0.062$). The comparison of the lowest four quintiles for the carcinoma-in-situ group with the highest gives an odds ratio of 4.0 which is also of borderline significance ($P=0.081$). When the two pre-invasive disease categories are combined an elevated odds ratio, again of borderline significance, is found for two of the three lower quintiles and for the four low quintiles combined (level I, $P=0.049$, level III, $P=0.021$, levels I–IV, $P=0.061$). Tests for

**Table I** Serum retinol and corrected $\beta$-carotene concentrations in control women and cases, by stage of disease of the cervix uteri.

Figures are arithmetic means ($\mu g l^{-1}$) for retinol and geometric means ($\mu g l^{-1}$) for $\beta$-carotene after age adjustment and shown with and without additional adjustment for current smoking, current OC usage, number of sexual partners, and social class.

| | | *Retinol* | | *β-carotene* | |
|---|---|---|---|---|---|
| | *N* | *Age adjusted*[1] | *Fully adjusted*[2] | *Age adjusted*[1] | *Fully adjusted*[2] |
| Severe dysplasia | 43 | 589.1 | 551.4 | 208.8[a] | 228.7 |
| Carcinoma-in-situ | 38 | 563.7 | 532.0 | 205.0[a] | 213.1[a] |
| All pre-invasive disease | 81 | 577.3 | 542.3 | 207.0[b] | 221.3[a] |
| Invasive cancer | 32 | 554.3 | 543.1 | 303.1 | 293.6 |
| Controls | 226 | 550.4 | 550.4 | 291.6 | 291.6 |

[a]Significantly different from control women, $P<0.05$;[3] [b]Significantly different from control women, $P<0.01$.

[1]Age adjusted means calculated by analysis of covariance (Armitage, 1971). The coefficients $a_i$ and $b_i$ are calculated after least squares fitting of the line:

$$\text{Serum concentration} = k + \sum_{i=1}^{11} a_i x_i + \sum_{i=1}^{3} b_i y_i$$

$x_i = 1$ when the subject is in the $(i+1)$th age stratum, else $x_i = 0$.
$y_1 = 1$ for case of severe dysplasia
$y_2 = 1$ for case of carcinoma in situ
$y_3 = 1$ for case of invasive cancer
else $y_i = 0$.

The adjusted means are then computed by substituting the proportion of controls in each age stratum for $x_i$ and setting $y_i = 0$ for the control means and $y_i = 1$ for the case means.

[2]Fully adjusted mean calculated as in (1) above but with extra terms in the model as follows:

$x_{12}$ = current smoking (0 = No, 1 = Yes)

$x_{13}$ = current OC usage (0 = No, 1 = Yes)

$x_{14}$ = No. of sexual partners (1 = 0 or 1, 2 = 2–5, 3 = 6+)

$x_{15}$ = Social class (1 = I, 2 = II, 3 = III, 4 = IV or V)

[3]Significance calculated from $t$-ratio (coefficient/standard deviation) for case-control terms in multiple regression model.

**Table II** Quintiles used in analysis and number in each quintile.

| | *Quintile* | | *Controls* | *Cases*: *Severe dysplasia* | *Carcinoma-in-situ* | *Invasive cancer* | *Total* |
|---|---|---|---|---|---|---|---|
| Retinol | | ($\mu g l^{-1}$) | | | | | |
| | I | 0–428 | 43 | 4 | 8 | 4 | 16 |
| | II | 429–501 | 46 | 9 | 9 | 9 | 27 |
| | III | 502–563 | 45 | 6 | 4 | 4 | 14 |
| | IV | 564–654 | 47 | 13 | 10 | 9 | 32 |
| | V (reference) | 655+ | 45 | 11 | 7 | 6 | 24 |
| | All | | 226 | 43 | 38 | 32 | 113 |
| β-carotene | | ($\mu g l^{-1}$) | | | | | |
| | I | 0–150 | 45 | 12 | 9 | 4 | 25 |
| | II | 151–245 | 47 | 8 | 11 | 9 | 28 |
| | III | 246–385 | 46 | 13 | 12 | 7 | 32 |
| | IV | 386–620 | 44 | 6 | 4 | 6 | 16 |
| | V (reference) | 620+ | 44 | 4 | 2 | 6 | 12 |
| | All | | 226 | 43 | 38 | 32 | 113 |

**Table III** Odds ratio calculations for quintile groups of serum retinol for different stages of cervical cancer (95% confidence intervals), after age stratification and adjustment for number of sexual partners, current smoking status, current usage of oral contraceptives, and social class.

| *Quintile* | *Severe dysplasia* | *Carcinoma-in-situ* | *All pre-invasive* | *Invasive cancer* |
|---|---|---|---|---|
| I–IV | 1.2 (0.5–2.8) | 2.0 (0.7–5.8) | 1.5 (0.7–3.1) | 1.5 (0.5–4.6) |
| I | 0.6 (0.2–2.3) | 1.9 (0.5–7.0) | 1.1 (0.4–3.0) | 1.1 (0.2–5.3) |
| II | 1.7 (0.5–5.3) | 2.3 (0.6–8.4) | 1.7 (0.7–4.5) | 2.1 (0.5–8.3) |
| III | 0.8 (0.2–2.7) | 1.1 (0.3–4.9) | 0.9 (0.3–2.6) | 1.1 (0.2–4.9) |
| IV | 1.7 (0.6–4.7) | 2.3 (0.7–7.9) | 2.1 (0.9–5.0) | 1.5 (0.4–5.6) |
| V | 1.0 | 1.0 | 1.0 | 1.0 |

**Table IV** Odds ratio calculations for quintile groups of serum $\beta$-carotene for different stages of cervical cancer (95% confidence intervals), after age stratification and adjustment for number of sexual partners, current smoking status, current usage of oral contraceptives, and social class.

| *Quintile* | *Severe dysplasia* | *Carcinoma-in-situ* | *All pre-invasive* | *Invasive cancer* |
|---|---|---|---|---|
| I–IV | 2.3 (0.7–7.3) | 4.0 (0.8–18.7) | 2.6 (1.0–7.1 ) | 1.3 (0.5–3.6) |
| I | 2.7 (0.7–11.4) | 5.3 (0.9–30.8) | 3.4 (1.0 – 11.2) | 1.1 (0.2–5.0) |
| II | 1.6 (0.4–6.5) | 4.4 (0.8–23.8) | 2.3 (0.7–7.2) | 1.4 (0.4–4.9) |
| III | 3.1 (0.9–11.6) | 4.9 (0.9–25.2) | 3.7 (1.2–11.3) | 1.7 (0.4–6.8) |
| IV | 1.9 (0.4–7.8) | 2.2 (0.3–13.6) | 1.7 (0.5–5.8) | 1.0 (0.3–3.7) |
| V | 1.0 | 1.0 | 1.0 | 1.0 |

trend in the odds ratios in the quintile groups just failed to reach statistical significance for the carcinoma-in-situ and both pre-invasive disease groups combined ($P = 0.052$ and $P = 0.088$, 2-sided, respectively).

We have also used these data to investigate the effects of smoking and oral contraceptive usage on retinol and $\beta$-carotene levels. The results of this analysis are shown in Table V for the control women only. Both smoking and OC usage are independently associated with the serum measures, both habits tending to be linked with relatively high retinol and low $\beta$-carotene levels respectively. The effect of smoking on $\beta$-carotene is quite strong, reducing the mean serum concentration by about 38%. These results were found to be similar in pre- and postmenopausal women.

### Discussion

Our findings do not support the idea that there is any association between low levels of serum retinol and an increased risk of cervical cancer. No significant relationship was found between the serum levels and any stage of disease. The findings do, however, suggest that $\beta$-carotene might have a weak inverse association with pre-invasive cancer although not with invasive cancer.

It is possible that the data for women with carcinoma-in-situ are the most informative, as women with invasive cancer may have had dietary and metabolic changes as a result of their disease (Wald *et al.*, 1986) while those with severe dysplasia may not all progress to invasive disease even if untreated. This was not, however, a prior hypothesis and the results, based on only 38 carcinoma-in-situ cases with large 95% confidence intervals, could be due to chance. The original epidemiological study (Harris *et al.*, 1980) was of pre-invasive disease only and a specific effect on such disease was postulated with the group of patients with invasive cancer added for comparison. It is, therefore, of interest that the results for pre-invasive disease show that cases have 25% lower $\beta$-carotene levels than controls and that not being in the top quintile for serum $\beta$-carotene appears to increase the risk of pre-invasive disease by about 2.6 times. In addition the trend between lower serum $\beta$-

**Table V** Age adjusted[a] serum retinol and corrected β-carotene concentrations in control women by current smoking and oral contraceptive use.

Figures are arithmetic means ($\mu g l^{-1}$) for retinol and geometric means ($\mu g l^{-1}$) for β-carotene.

| | *Non-current smoker* | *Current smoker* | *All* | *Significance of association with smoking* |
|---|---|---|---|---|
| Non-current OC user | | | | |
| No. | 112 | 54 | 166 | |
| Serum retinol | 509.8 | 564.8 | 527.7 | <0.05 |
| Serum β-carotene | 367.3 | 229.6 | 314.2 | <0.001 |
| Current OC user | | | | |
| No. | 37 | 23 | 60 | |
| Serum retinol | 592.7 | 648.2 | 612.9 | NS |
| Serum β-carotene | 281.6 | 176.0 | 237.3 | <0.5 |
| All | | | | |
| No. | 149 | 77 | 226 | |
| Serum retinol | 530.7 | 588.3 | 550.4 | <0.01 |
| Serum β-carotene | 343.0 | 213.1 | 291.6 | <0.001 |
| Significance of association with OC use | | | | |
| Retinol | <0.5 | <0.05 | <0.001 | |
| β-carotene | NS | NS | <0.05 | |

[a]Adjusted by analysis of covariance as in footnote (1) **Table I.**

carotene and increased risk of pre-invasive disease is of borderline significance.

Cervical cancer has been shown to be associated with a number of risk factors, those relating to sexual behaviour having by far the largest effect (Harris *et al.*, 1980; Singer, 1979). Low social class (OPCS, 1982), smoking (Greenberg *et al.*, 1985), and possibly long-term oral contraceptive usage (Harris *et al.*, 1980; Peritz *et al.*, 1977) are, however, also positively associated with this disease. Within this context of a multifactorial aetiology and taking current knowledge of the effects of vitamin A and related substances into account. it seems unlikely that more than a weak inverse association between vitamin intake and cancer incidence would be found. Our results for pre-invasive cancer and β-carotene are consistent with such a relationship, but even a weak relationship, if causal, could indicate a method of prophylaxis which would have a major effect on cancer incidence rates (Peto, 1983). Prophylaxis with β-carotene would moreover be practicable as increased consumption is directly and proportionally reflected in increased serum levels, which is not true of retinol as the serum level is homeostatically maintained more or less constant and excessive consumption can be hepato-toxic. It might therefore be profitable to investigate further the specific association between β-carotene and carcinoma-in-situ.

One previous study has found an inverse association between severe dysplasia and carcinoma in situ and dietary consumption of foods rich in β-carotene (Romney, *et al.*, 1981) while two have found a similar relationship with invasive cancer (Marshall *et al.*, 1983; La Vecchia *et al.*, 1984). None of them have found any association with retinol consumption. Of the three other serum studies, the two concerned with pre-invasive disease (Bernstein & Harris, 1984; Lambert *et al.*, 1981) showed no significant effects whereas the third (Orr *et al.*, 1985) showed that women with invasive disease had lower β-carotene, but not retinol, levels than controls.

Our results are consistent with those of most other studies which have looked at the influence of smoking and oral contraceptive usage on serum retinol and β-carotene level. The tendency for smoking to be associated with an increased retinol and decreased β-carotene level has been reported previously (Yeung, 1976; Witter *et al.*, 1982; Salonen *et al.*, 1985) although some data sets are not consistent with such a relationship for retinol (Wald, personal communication). A positive effect of OC usage on retinol concentrations has also been found previously Gal *et al.*, 1971; Gal & Parkinson, 1973; Yeung, 1974, 1976; Smith *et al.*, 1975; Yeung & Chan, 1975), but there is some controversy as to whether it affects β-carotene (Yeung & Chan, 1975).

The general conclusions from other studies – that smoking is more effective than OC usage in reducing $\beta$-carotene and less effective in increasing retinol – are, however, confirmed by our results. It is unclear whether the mode of action is the result of a direct pharmacological effect (perhaps catalysing the conversion of $\beta$-carotene to retinol or stimulating the synthesis of retinol binding protein and hence increasing serum concentration) or whether smoking and OC usage tend to be associated with dietary habits that lead to a reduced $\beta$-carotene intake and increased retinol circulation. The fact that serum vitamin A levels fluctuate in a cyclical pattern throughout the menstrual cycle (Yeung, 1974), and that this pattern is disturbed by OC usage (Gal *et al.*, 1971; Gal & Parkinson, 1973; Yeung, 1974), would suggest a direct, if complex, biochemical relationship between hormonal and micronutrient components in the serum. It is of course possible that both biochemical effects and associated dietary changes accompanying OC usage and smoking are involved.

Since smoking and OC usage affect retinol and $\beta$-carotene and our previous report (Harris *et al.*, 1980) also associated these variables with the risk of precancerous lesions, they are both potential confounding factors. Indeed it is possible that these variables affect disease through the modification of micronutrient levels. They were, therefore, both taken into account in our analysis of odds ratios, as were number of sexual partners and social class. Number of sexual partners was a major risk factor in our previous analysis and although social class was not a risk factor in this study it has been reported as such in many other investigations (OPCS, 1982). Neither of these factors affected retinol levels in our control series but there was a relationship between social class and $\beta$-carotene concentration, which was higher in women in the upper social class groups.

The authors would like to thank Drs R.M. Salkeld and J.P. Vuilleumier, Hoffman-La Roche, Basle, for carrying out the biochemical analyses. Dr M.C. Pike helped us with statistical advice and by improving previous drafts of the manuscript. We also thank Miss C. Bates for typing successive drafts of the paper.

### References

ARMITAGE, P. (1971). *Statistical Methods in Medical Research*, Blackwell Scientific Publications: Oxford.

BERNSTEIN, A. & HARRIS, B. (1964). The relationship of dietary and serum vitamin A to the occurrence of cervical intraepithelial neoplasm in sexually active women. *Am. J. Obstet. Gynecol.* **148**, 309.

BJELKE, E. (1975). Dietary vitamin A and human lung cancer. *Int. J. Cancer*, **15**, 561.

BOLLAG, W. (1979). Retinoids and cancer. *Cancer Chemother. Pharmacol.* **3**, 207.

BRESLOW, N.E. & DAY, N.E. (1980). Statistical methods in cancer research. *IARC Scientific Publications* No.32. IARC: Lyon.

GAL, I., PARKINSON, C. & CRAFT, I. (1971). Effects of oral contraceptives on human plasma vitamin A levels. *Brit. Med. J.*, **2**, 436.

GAL, I. & PARKINSON, C. (1973). Changes in serum vitamin A levels during and after oral contraceptive therapy. *Contraception*, **8**, 13.

GREENBERG, E.R., VESSEY, M., McPHERSON, K. & YEATES, D. (1985). Cigarette smoking and cancer of the uterine cervix. *Br. J. Cancer*, **51**, 139.

HAINES, A.P., THOMPSON, S.G., BASU, T.K. & HUNT, R. (1982). Cancer, retinol binding protein, zinc and copper. *Lancet* **i**, 52.

HARRIS, R.W.C., BRINTON, L.A., COWDELL, R.H. & 4 others (1980). Characteristics of women with dysplasia or carcinoma in situ of the cervix uteri. *Br. J. Cancer*, **42**, 359.

HIRAYAMA, T. (1979). Diet and cancer. *Nutr. Cancer*, **1**, 67.

JOHNSON, L.D. (1969). The histopathological approach to early cervical neoplasia. *Obstet. Gynecol. Survey*, **24**, 735.

KARK, J.D., SMITH, A.H., SWITZER, B.R. & HAMES, C.G. (1981). Serum vitamin A (retinol) and cancer incidence in Evans County, Georgia. *J. Natl Cancer Inst.*, **66**, 7.

KVÅLE, G., BJELKE, E. & GART, J.J. (1983). Dietary habits and lung cancer risk. *Int. J. Cancer*, **31**, 397.

LA VECCHIA, C., FRANCESCHI, S., DE CARLI, A. & 4 others (1984). Dietary vitamin A and the risk of invasive cervical cancer. *Int. J. Cancer*, **34**, 319.

LAMBERT, B., BRISSON, G. & BIELMAN, P. (1981). Plasma vitamin A and precancerous lesions of cervix uteri: a preliminary report. *Gynecol. Oncol.*, **11**, 136.

MARSHALL, J.R., GRAHAM, S., BYERS, T., SWANSON, M. & BRASURE, J. (1983). Diet and smoking in the epidemiology of cancer of the cervix. *J. Natl Cancer Inst.*, **70**, 847.

MATHEWS-ROTH, M.M. & STAMPFER, M.J. (1984) Some factors affecting deterioration of carotenoids in serum. *Clin. Chem.*, **30**, 459.

METTLIN, C. & GRAHAM, S. (1979). Dietary risk factors in human bladder cancer. *Am. J. Epidemiol.*, **110**, 255.

METTLIN, C., GRAHAM, S. & SWANSON, M. (1979). Vitamin A and lung cancer. *J. Natl Cancer Inst.*, **62**, 1435.

NOMURA, A.M.Y., STEMMERMANN, G.N., HEILBRON, L.K., SALKELD, R.M. & VUILLEUMIER, J.P. (1985). Serum vitamin levels and the risk of cancer of specific sites in men of Japanese ancestry in Hawaii. *Cancer Res.*, **45**, 2369.

OFFICE OF POPULATION CENSUSES AND SURVEYS (1982). Cancer Mortality by Occupation and Social Class. *SMPS* No. 44, HMSO: London.

ORR, J.W., WILSON, K., BODIFORD, C. & 5 others (1985). Nutritional status of patients with untreated cervical cancer. *Am. J. Obstet. Gynecol.*, **151**, 632.

PELEG, I., HEYDEN, S., KNOWLES, M. & HAMES, C.G. (1984). Serum retinol and risk of subsequent cancer: extension of the Evans County, Georgia study. *J. Natl Cancer Inst.*, **73**, 1455.

PERITZ, E., RAMACHARAN, S., FRANK, J., BROWN, W.L., HUONG, S. & RAY, R. (1977). The incidence of cervical cancer and duration of oral contraceptive use. *Am. J. Epidemiol.*, **106**, 462.

PETO, R. (1983). The marked differences between carotenoids and retinoids: methodological implications for biochemical epidemiology. *Cancer Surveys*, **2**, 327.

PETO, R., DOLL, R. BUCKLEY, J.D. & SPORN, M.B. (1981). Can dietary beta-carotene materially reduce human cancer rates? *Nature*, **290**, 201.

RAPHAEL, S.I. & WATERMAN, G.W. (1951). Cancer of the uterine cervix. *N. Engl. J. Med.*, **245**, 281.

ROMNEY, S.L., PALAN, P.R., DUTTAGUPTA, C. & 5 others (1981). Retinoids and the prevention of cervical dysplasias. *Am. J. Obstet. Gynecol.*, **141**, 890.

SALONEN, J.T., SALONEN, R., LAPPATELAINEN, R., MÄENPÄÄ, P.H., ALFTHAN, G. & PUSKA, P. (1985). Risk of cancer in relation to serum concentrations of selenium and vitamins A and E: matched case-control analysis of prospective data. *Br. Med. J.*, **290**, 417.

SHEKELLE, R.B., LEPPER, M., LUI, S. & 6 others (1981). Dietary vitamin A and risk of cancer in the Western Electric Society. *Lancet*, **ii**, 1185.

SINGER, A. (1979). Further evidence for high risk male and female groups in the development of cervical carcinoma. *Obstet. Gynecol. Surv.*, **34**, 867.

SMITH, J.L., GOLDSMITH, G.A. & LAWRENCE, J.D. (1975). Effects of oral contraceptive steroids on vitamin and lipid levels in serum. *Am. J. Clin. Nutr.*, **28**, 371.

STAHELIN, H.B., BUESS, E., RÖSEL, F., WIDMER, L.K. & BRUBACHER, G. (1982). Vitamin A, cardiovascular risk factors and mortality. *Lancet*, **i**, 394.

VUILLEUMIER, J.P., KELLER, H.E., GEYSEL, D. & HUNZIKER, F. (1983). Clinical chemical methods for the routine assessment of the vitamin status in human populations. Part 1. The fat-soluble vitamins A and E, and beta-carotene. *Int. J. Vit. Nutr. Res.*, **53**, 265.

WALD, N., IDLE, M., BOREHAM, J. & BAILEY, A. (1980). Low serum vitamin A and subsequent risk of cancer. *Lancet*, **ii**, 813.

WALD, N.J., BOREHAM, J., HAWYARD, J.L. & BULBROOK, R.D. (1984). Plasma retinol, beta-carotene and vitamin E levels in relation to the future risk of breast cancer. *Br. J. Cancer*, **49**, 321.

WALD, N.J., BOREHAM, J. & BAILEY, A. (1986). Serum retinol and subsequent risk of cancer. Submitted for publication.

WILLETT, W.C., POLK, B.F., UNDERWOOD, B.A. & 6 others (1984). Relations of serum vitamin A and E and carotenoids to the risk of cancer. *N. Engl. J. Med.*, **310**, 430.

WILLETT, W.C., POLK, B.F. UNDERWOOD, B.A. & HAMES, C.G. (1984). Hypertension detection and follow-up program study of serum retinol, retinol-binding protein, total carotenoids, and cancer risk: A summary. *J. Natl Cancer Inst.*, **73**, 1459.

WITTER, F.R., BLAKE, D.A., BAUMGARDNER, R., MELLITS, E.D. & NIEBYL, J.R. (1982). Folate, carotene and smoking. *Am. J. Obstet. Gynecol.*, **144**, 857.

YEUNG, D.L. (1974). Effects of oral contraceptives on vitamin A metabolism in the human and the rat. *Am. J. Clin. Nutr.*, **27**, 125.

YEUNG, D.L. (1976). Relationships between cigarette smoking, oral contraceptives and plasma vitamins A, E, C, and plasma triglycerides and cholesterol. *Am. J. Clin. Nutr.*, **29**, 1216.

YEUNG, D.L. & CHAN, P.S. (1975). Effects of a progestogen and sequential type oral contraceptive on plasma vitamin A, vitamin E, cholesterol and triglycerides. *Am. J. Clin. Nutr.*, **28**, 686.